# The Potential of Optical Profilometry in the Study of Cultural Stone Weathering

**DOI:** 10.3390/jimaging5060060

**Published:** 2019-06-16

**Authors:** Dario Ambrosini, Tullio de Rubeis, Iole Nardi, Domenica Paoletti

**Affiliations:** 1 Department of Industrial and Information Engineering and Economics, University of L’Aquila, P.le Pontieri 1, I-67100 L’Aquila, Italy; 2 ISASI-CNR, Institute of Applied Science and Intelligent Systems, Via Campi Flegrei 34, I-80078 Pozzuoli (NA), Italy; 3 ENEA-Casaccia Research Center, Via Anguillarese, 301, S.M. di Galeria, I-00123 Roma, Italy

**Keywords:** weathering, optical profilometry, artwork inspection, cultural stone

## Abstract

The problem of deterioration of marble or stone monuments on display in the open air was raised in scientific terms around the mid-nineteenth century, correctly sensing the close dependence between the increased speed of surfaces alteration and air pollution. However, only more recently, around the years 1980–1990, emerged a need for quantitative data to assess the degree of degradation and the relative danger in the future projections. Non-destructive techniques can be an important aid in assessing the state of degradation and, above all, its speed, directly on the most important monuments exposed to the urban environment. In this work we discuss some non-destructive techniques able to evaluate the alteration of the surface shape of artefacts exposed to the environment through a non-contact survey of their surface shape. Advantages and disadvantages will be highlighted, as well as the problems still open.

## 1. Introduction

Recent decades have seen the growth of a generalized interest, both of the scientific community and of public opinion, in the protection and conservation of cultural heritage. Despite this interest, and sometimes because of it, works of art are more than ever at risk. This is particularly true of drawings, paintings and frescoes, but also apparently “resistant” artefacts, such as those made of stone, are in danger.

Stone degradation is a natural phenomenon, to which physical, chemical and biological processes contribute. The extent of degradation depends in a complex way on the nature of the constituent materials and on their exposure to a particular environment [1,2,3,4,5].

**Figure 1:**
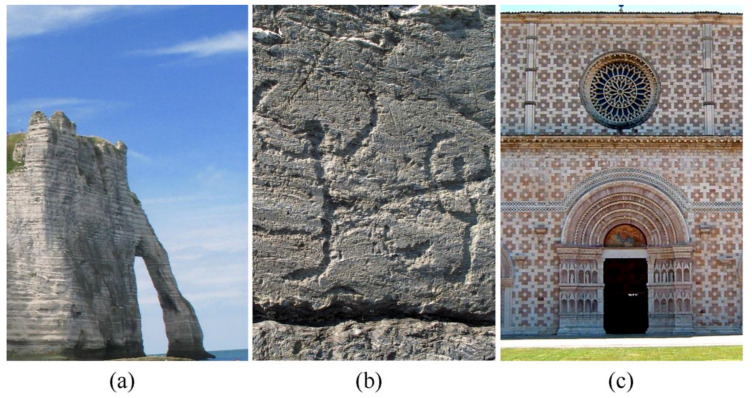
Terminology in stone decay research; (**a**) rock (chalk cliffs, Étretat, France); (**b**) cultural rock (Rupe Magna, rock engraving park of Grosio, Italy); (**c**) building stone (Santa Maria di Collemaggio, XIII century, L’Aquila, Italy). Both (b) and (c) can be referred to as cultural stone.

Stone used in human artefacts, as a building material or processed to obtain works of art (statues, bas-reliefs, etc.), is sometimes referred to in literature as cultural stone to distinguish it from natural stone, see Figure 1. Although a common language is lacking, natural stone, not deliberately altered by humans, is usually referred as rock. A rock, staying in its original environment, but showing traces of human art, is referred as cultural rock. When rock is removed from the original environment to realize cultural heritage, the term building stone can be used.

The term cultural stone was introduced for a “stone that has been physically altered by humans—abraded, engraved, quarried, chipped or chiseled, or dressed” [6]. 

Cultural stone is subject to an even greater number of degradation factors such as, in general, exposure to polluted environments (urban, industrial), damage suffered during the phases of extraction and/or processing, choice of materials or treatments inappropriate for the purpose and/or the environment of destination. 

A famous example is the Obelisk of Cleopatra, in New York, whose degradation has been greatly accelerated by the shift from an arid to a humid climate and by a treatment with wax, to protect it from the elements, which has also sealed inside the saline moisture [7,8].

The problem of deterioration of marble or stone monuments outdoors was raised in scientific terms around the mid-nineteenth century, correctly sensing the link between the increased speed of surface alteration and air pollution. But air pollution is not just a modern phenomenon: there are ancient observations (Greeks, Romans, 17th–18th centuries) of the deterioration of monuments [9], as well as evidence of pollution by wood fires in Italian and French medieval and Renaissance cities (Bologna, Arles, Paris) [10] and by the habit of burning coal, which has spread in London since the 13th century [11].

The study of the degradation of stone (and the methods to contain it) must necessarily be interdisciplinary, including disciplines such as chemistry, physics, materials science, engineering, geomorphology and those involved in artwork conservation [1,2,3,4,5,6,7,8,9,10,11]. Research first focused on the stone–pollution interaction; only more recently, around the years 1980–1990, emerged a need for quantitative data to assess the degree of degradation and its danger in future projections.

Stone degradation, as mentioned above, is a very complex phenomenon, the mechanisms of which certainly requires further study. In any case, the final effect of the various damage factors is generally the disintegration of the stone itself. Therefore, an average overall parameter indicative of the extent of the degradation itself is represented by the loss of material, i.e., the surface recession. 

This has been a line of research that has been very much followed: if the “zero level”, from which the degradation starts, is known and if the loss of material is measured, then the rate of corrosion, generally expressed as a loss of thickness in mm per century, can be evaluated.

Two fundamental approaches were used for these studies: the evaluation of the loss of material on stone structures whose time of exposure to the environment is known (tombstones, buildings) and the exposure to real environments of slabs of materials specially made. The determination of weathering rates is difficult; typical measurements of rates of sandstone erosion are in the 0.007–0.7 mm/year range [12]. Some studies suggest that the rate of degradation decreases over time [13,14], others, on the contrary, suggest that it increases [15,16]. Local factors are important, as well as a kind of threshold behavior for the rate of degradation [6].

In conclusion, there is still much to be done to understand the phenomenon of stone degradation and to be able to evaluate it quantitatively in a satisfactory way. In any case, as the loss of material per year is typically in the range of μm, non-destructive optical techniques, having a sensitivity in the same range, can represent suitable tools to assess weathering or stone decay.

In this work we discuss some non-destructive techniques able to evaluate the alteration of the surface shape of artefacts exposed to the environment through a non-contact survey of their surface shape. Advantages and disadvantages will be highlighted, as well as the problems still open.

## 2. Optical Techniques for Assessing Surface Degradation and Material Loss

The accurate measurement of the three-dimensional shape of an object is born in the industrial field for production and quality control needs and currently has multiple applications (robotics, anthropometry, reverse engineering, etc.). The rapid evolution of hardware and software tools (CAD modeling, image processing) and the “digital revolution” have led to the spread of increasingly powerful and versatile techniques, which can give an important contribution in the field of Cultural Heritage. Typical applications range from the diagnosis of defects, to the replication of museum objects, real or virtual [17,18,19].

It has been seen how, in general, the degradation of stone translates into a modification of its surface. Therefore, a precise assessment of the extent of the damage can be obtained by comparing the shape of the surface with its initial condition (if available). In addition, the deterioration rates could be obtained by a constant monitoring.

This is one of the approaches generally followed in material loss studies in conjunction with the assessment of surface roughness [6].

Another important aspect to consider is the portability of the technique: if the measurement is made on samples, the whole procedure takes place in the laboratory, if the measurement is to be made in situ, the used technique must have specific requirements.

Good methods for assessing weathering degradation should have most of the following features:

1. Possibility of use in situ;
2. Low sensitivity to external disturbances;
3. Good measurement sensitivity;
4. Low cost and simplicity of the system;
5. Possibility of easy use even by operators not specialized in non-destructive techniques;
6. Full digital and automated data processing.

### 2.1. Holographic Contouring

Holographic interferometry (HI) can be used to obtain a three-dimensional map of the object in terms of contour lines [20]. This very sensitive technique gives high quality images, but it suffers from all the disadvantages of holography: basically, operational difficulties, poor portability and high costs. Therefore, it will be considered in the following only for sake of comparison.

### 2.2. ESPI

**Figure 2:**
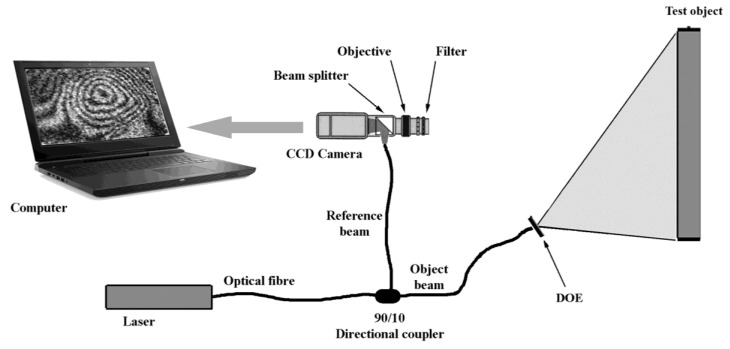
A typical experimental setup for portable electronic speckle pattern interferometry (ESPI). It is also suitable to perform fringe projection profilometry.

Electronic speckle pattern interferometry (ESPI), also called digital speckle pattern interferometry (DSPI) is an experimental technique like holographic interferometry but designed to overcome its operational difficulties [21,22]. A laser source, a camera with its acquisition card and a computer are needed to manage the system (Figure 2); the use of optical fibers enhances portability, while the filter, centered on the laser wavelength, allows working in ambient light. When performing ESPI, the diffractive optical element (DOE) must be removed.

When a surface is illuminated by laser light it shows a typical graininess (commonly called a speckle pattern). The average grains size is inversely proportional to the relative aperture of the lens, which must therefore be small enough to allow their resolution by the camera. Inside the lens (and before the camera sensor, typically a charge-coupled device -CCD) there is a beam splitter (normally consisting of two glued prisms) that superimposes on the CCD the image of the object with an undisturbed beam of light, called the reference beam. It can be demonstrated that this superimposed image contains information on the phase and therefore on all the quantities that can modify it, such as temperature, vibrations or the profile of the surface under examination, according to different possible applications.

The ESPI technique was used for the diagnosis of defects [23,24,25,26] and for the measurement of the surface profile [27,28]. Computer processing produces holographic-like (but noisier) fringes that can be further processed to obtain the surface profile point by point.

As far as portability is concerned, any relative movement, in the order of the wavelength of the laser used, between the optical system and the object under test induces unwanted fringes that inevitably alter the test result. During field operation, the presence of mechanical vibrations is the main source of disturbance (uncontrollable micro displacements). A simple and efficient solution consists in the realization of a rigid mechanical coupling between the measuring sensor and the object under test. This solution is not always feasible when you want to study art, furthermore it greatly reduces the flexibility of the system.

An alternative solution may be to assemble the interferometric sensor on a “cabinet”. The tripod plus the interferometric sensor must be made in such a way as to be as insensitive as possible to external vibrations. 

### 2.3. Fringe Projection

The projection of a system of fringes on a surface is a very common method for obtaining the profile. The operating principle is based on the fact that the lines follow the profile and therefore provide quantitative information about it. There are many examples of different experimental arrangements in the literature. Here we will briefly describe some of the experiences developed at the Engineering School in L’Aquila.

A first version (hereinafter called FP1) uses the interference of light from two optical fibers for the generation of fringes [29,30]. This system requires a laser, a pair of optical fibers and the usual acquisition camera. The projected fringes are sinusoidal, which brings some advantages in terms of noise during image processing. It is a simple and economical system with good resistance to possible mechanical disturbance vibrations.

A second version (FP2) uses black and white lines (Ronchi’s grid) projected on the surface with white light and therefore requires neither lasers nor optical fibers. This system is cheaper and exhibits less noise than the FP1 [31].

Finally, the third version (FP3) still uses an interference phenomenon based on a diffraction grating and a small laser diode. This system is characterized by an extreme compactness that makes it very robust and portable and theoretically suitable also for a fixed positioning in situ [32]. Figure 2 shows how FP3 can be incorporated in an ESPI system: when performing fringe projection, the DOE must be inserted, and the reference beam must be blocked. For detailed information, the reader is referred to the original publication [32].

### 2.4. Conoscopic Holography

Conoscopic holography (CH) is a technique [33,34,35] that uses the properties of birefringent crystals to make holograms with some salient features: it is possible to use almost monochromatic although not spatially coherent sources, has great stability and easy interfacing with a computer.

It is a very powerful and sensitive technique, but its precise measurements are not full field, so it is necessary to scan the object under investigation. A further advantage is the availability of commercial systems. In the field of Cultural Heritage, conoscopic holography was used, for example, to determine the profile of ancient coins [36] and for measuring surface topography [37]. Given its high sensitivity, it can be used for quantitative determinations of the loss of material through measurements of the surface profile or its roughness, as proposed in [38,39,40]. Thanks to the availability of commercial systems with dedicated software, conoscopic holography could become the reference system for the measurement of profiles (and therefore also of loss of material) in the laboratory.

## 3. Results and Discussion

The presented results illustrate the potential of non-destructive techniques in the determination of profiles and material loss.

**Figure 3:**
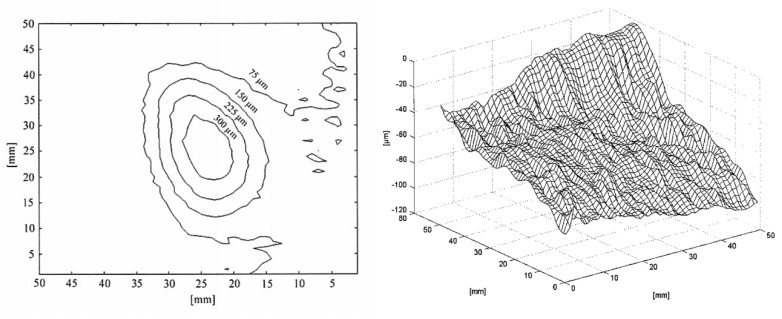
Contour map of an eroded area on a limestone sample (**left**) [29] and 3D plot of material loss by an artificial erosion on marble (**right**) [30].

Figure 3 (left) shows the application of the FP1 method on a limestone sample exposed to an acid spray (diluted sulfuric acid solution) to simulate erosion in laboratory.

Figure 3 (right) shows the difference between the original surface profile and the profile after 10 minutes exposure to an acid spray: thus, the volume between the zero-height plane and the surface profile represents the volume of the lost material.

**Figure 4:**
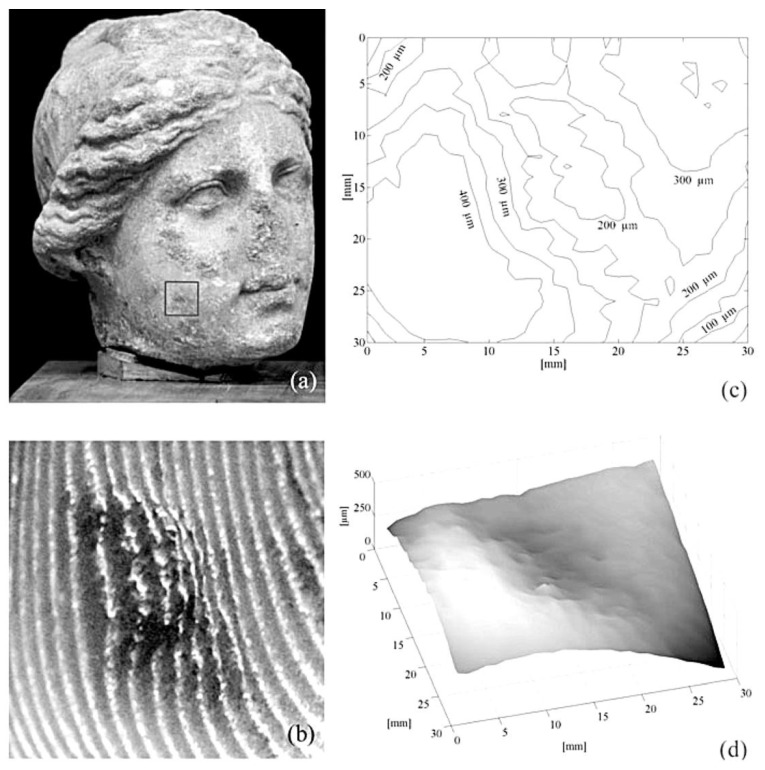
(**a**) Stone head of Roman age; (**b**) projected fringe, (**c**) contour plot, (**d**) 3D plot of the surface, on the selected area of (a) [30].

Figure 4 shows an example of application of the projection fringe method FP1 on a real artwork from the Museo Nazionale d’Abruzzo (L’Aquila, Italy). The performance of the FP2 and FP3 methods are like FP1; main differences lie in compactness and flexibility.

**Figure 5:**
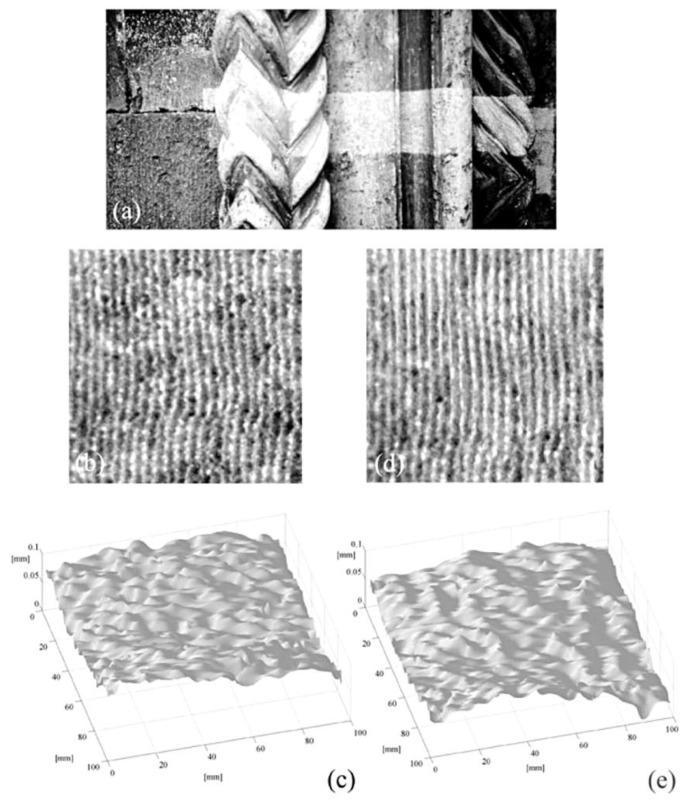
Investigation of the stone portal of the Cathedral of Atri (Italy) during laser cleaning; (**a**) laser cleaning in progress on a detail of the portal; (**b**) contour fringes before cleaning; (**c**) 3D plot obtained from (b); (**d**) contour fringes after cleaning; (**e**) 3D plot obtained from (d), [30].

Figure 5 shows an example of measurements performed on the stone portal of the Atri Cathedral (XIII century). A comparison between Figure 5c,e proves that the cleaning action preserves the surface texture (same mean roughness).

Finally, Table 1 briefly compares the techniques examined, from the point of view of sensitivity, portability and cost. Portability and cost have been described qualitatively, with respect to HI.

## 4. Conclusions

In the Introduction we have seen how the complex phenomenon of stone degradation translates, in any case, into surface deterioration, with relative loss of material quantifiable in the μm/year range.

Non-destructive techniques based on optical methods have sensitivities in this range and therefore deserve to be analyzed.

In this work we have briefly considered the following techniques: holographic contouring, ESPI, projected fringes in three different measurement configurations and finally conoscopic holography. The techniques have different features, summarized in Table 1.

Let us start by observing that holographic contouring, even if very sensitive, does not seem a viable choice. In fact, it is a technique that is difficult to use and that presents high costs and very poor portability. 

ESPI, as widely discussed in the literature, is a major step forward. It has a similar sensitivity to holography and good portability. Costs are significantly lower than holography, but some operational difficulties remain. It should also be borne in mind that ESPI has a much lower image quality than holography and therefore generally requires digital processing, often dedicated, to extract the information of interest. Finally, the typical sensitivity indicated depends on a set of experimental parameters. In general, we can say that the maximum sensitivities (in the order of the values in Table 1) can be reached on relatively small areas.

Conoscopic holography can be considered the reference benchmark for laboratory measurements. It has a sensitivity of less than μm and, in addition, fully integrated measurement systems that can also be used by personnel who are not experts in non-destructive techniques, are commercially available. The only obstacles are the cost, still relatively high, and the measurement time that, requiring scanning of the sample, can be long in the case of measurements with maximum sensitivity.

The projected fringes techniques, on the other hand, represent the best option as far as portability and costs are concerned, therefore the most recommended choice to try to carry out in situ monitoring. The three versions discussed have practically the same sensitivity (which depends on the number of projected fringes). The FP3 method was considered superior because it is very compact and robust. Moreover, it is very easy to change the number of fringes.

The possibility of successfully using these techniques to monitor the stone deterioration in situ, directly on monuments, however, is still an open problem. Weathering is a slow process, in the order of μm/year for limestone, and can be episodic, causing flaking and scaling rather than disintegration or dissolution, for cultural stone in polluted environments.

In any case, since stone decay is not uniform, the relative change in elevation of the surface by material loss can be monitored with respect to a reference point (i.e., an unweathered point) on the surface.

## Figures and Tables

**Table 1:** Rough comparison of the techniques.

Technique	Sensitivity	Portability	Cost
HI	≈ 1 μm	poor	high
ESPI	≈ 1 μm	good	medium
FP1	≈ 5 μm	good	low
FP2	≈ 5 μm	good	low
FP3	≈ 5 μm	very good	low
CH	≈ 0.1 μm	medium–good	medium
